# Putative Core Transcription Factors Affecting Virulence in *Aspergillus flavus* during Infection of Maize

**DOI:** 10.3390/jof9010118

**Published:** 2023-01-14

**Authors:** Matthew K. Gilbert, Brian M. Mack, Matthew D. Lebar, Perng-Kuang Chang, Stephanie R. Gross, Rebecca R. Sweany, Jeffrey W. Cary, Kanniah Rajasekaran

**Affiliations:** Agricultural Research Service, United States Department of Agriculture, 1100 Allen Toussaint Blvd, New Orleans, LA 70124, USA

**Keywords:** *Aspergillus flavus*, HacA, virulence, oxidative stress, host resistance, cyclopiazonic acid

## Abstract

*Aspergillus flavus* is an opportunistic pathogen responsible for millions of dollars in crop losses annually and negative health impacts on crop consumers globally. *A. flavus* strains have the potential to produce aflatoxin and other toxic secondary metabolites, which often increase during plant colonization. To mitigate the impacts of this international issue, we employ a range of strategies to directly impact fungal physiology, growth and development, thus requiring knowledge on the underlying molecular mechanisms driving these processes. Here we utilize RNA-sequencing data that are obtained from in situ assays, whereby *Zea mays* kernels are inoculated with *A. flavus* strains, to select transcription factors putatively driving virulence-related gene networks. We demonstrate, through growth, sporulation, oxidative stress-response and aflatoxin/CPA analysis, that three *A. flavus* strains with knockout mutations for the putative transcription factors AFLA_089270, AFLA_112760, and AFLA_031450 demonstrate characteristics such as reduced growth capacity and decreased aflatoxin/CPA accumulation in kernels consistent with decreased fungal pathogenicity. Furthermore, AFLA_089270, also known as HacA, eliminates CPA production and impacts the fungus’s capacity to respond to highly oxidative conditions, indicating an impact on plant colonization. Taken together, these data provide a sound foundation for elucidating the downstream molecular pathways potentially contributing to fungal virulence.

## 1. Introduction

*Aspergillus flavus* is a saprophytic and opportunistic phytopathogenic fungus that infects several important food and feed crops such as maize, peanut, and treenuts. After colonizing the plant, *A. flavus* has the potential to produce several toxic secondary metabolites (SMs) including the structurally similar aflatoxins (AFs), the most toxic and carcinogenic of which is aflatoxin B_1_ (AFB1) [1]. Among the most impacted of crops is maize, which is grown worldwide and is susceptible to *A. flavus* infection, especially during seasonal high temperatures and reduced water availability [2]. It has been reported that maize crop losses due to AF contamination in the U.S alone have reached USD 686.6 million per year [3]. Potential changes in climate conditions could exacerbate the impact of aflatoxins in maize with some estimates as high as USD 1.68 billion per year [3].

Mitigation efforts to control pre-harvest aflatoxin contamination in maize include classical breeding to select for *A. flavus* resistance [4] and biocontrol approaches involving the application of non-aflatoxigenic *A. flavus* spore formulations onto developing crops in the field, which are thought to then displace native toxigenic strains, thus reducing overall contamination levels [5,6]. Recent transgenic approaches involve the use of host-induced gene silencing (HIGS) through RNA interference (RNAi) technology [7,8]. Here, specific mRNAs in the colonizing pathogen are targeted by small interfering RNA (siRNA) molecules produced by the transgenic host crop, resulting in the target genes subsequent RNAi-induced degradation. While the use of this technology has several benefits over traditional mitigation strategies, such as multi-gene targeting and a semi-broad application to a range of species [9,10], the identification and selection of target genes is an obviously crucial component, with emphasis being on virulence factors including transcription factors that regulate multiple virulence-related processes. Here we refer to transcription factors essential for high levels of pathogenicity as core transcription factors. 

RNA-sequencing of pathogen-susceptible and -resistant maize kernels undergoing infection has provided insight into flavonoid-pathway genes in maize related to host resistance [11]. In this study, using the same experimental basis, we conducted bioinformatic analysis of fungal genes during maize infection to identify core transcription factors potentially driving virulence-related gene networks. We identified significantly differentially expressed transcription factor genes and generated knockout mutants of selected candidate genes for further analysis. The mutant strains were assayed for development, aflatoxin and cyclopiazonic acid production, and response to oxidative stress. 

## 2. Materials and Methods

### 2.1. Fungal Strains and Growth Conditions 

For RNA sequencing, *A. flavus* strain NRRL 3357 [12] was grown at 31 °C on V8 medium (5% V8 Vegetable Juice (Campbell Soup Company, Camden, NJ, USA), 2% agar, pH 5.2). The spore inoculum was prepared from 6-day old cultures suspended in 0.02% Triton X-100; the spore concentration was determined with a hemocytometer and adjusted to 4 × 10^6^ spore mL^−1^. The maize lines used for the kernel infection assay (KIA) and for RNA sequencing were Va35 (NPGS Acc. PI 587150), TZAR102 (NPGS Acc. PI 654049) [4,13], and MI82 [13]. For generating knockouts, *A. flavus* strain CA14 was grown on V8 medium described above [14]. Yeast, glucose, trace element (YGT) agar was made according to Kafer [15]. 

### 2.2. Kernel Infection Assays 

Two separate KIAs were conducted, both based on modification of the kernel screening assay (KSA) described previously by Gilbert et al. [8] and Castano-Duque et al. [11]. The first KIA to obtain material for RNA sequencing was conducted as described [11]. Briefly, healthy kernels were sterilized with 70% ethanol and soaked in an inoculum of 4 × 10^6^ fresh spores/mL of *A. flavus* NRRL 3357 for 5 min. Kernels were then incubated at 25 °C under 12 h light/dark cycles for nine days. The second KIA for UPLC analysis was conducted in a similar manner, except the susceptible maize line B73 [8] was inoculated with *A. flavus* CA14 and the transcription factor knockout strains described below. All KIA and subsequent assays were conducted in triplicate.

### 2.3. RNA Extraction and Library Preparation for RNA Sequencing 

RNA extraction was conducted as described in Castano-Duque et al. [11]. Briefly, kernel samples were ground with 0.5 mm diameter zirconia-silica beads (BioSpec Products, Bartlesville, OK, USA) using a TissueLyser II (Qiagen, Germantown, MD, USA). Total RNA was extracted using QIAzol Lysis Reagent (Qiagen) following the miRNeasy Mini Kit manufacturer’s protocol (Qiagen). For DNase treatment, PureLink DNase (ThermoFisher, Waltham, MA, USA) was used. RNA quality was confirmed using a 2100 Bioanalyzer with the 6000 Nano Kit (Agilent, La Jolla, CA, USA). A total of 1 μg of RNA was used to do further DNase treatment using TURBO DNase (Thermofisher). mRNA was then isolated with Dynabeads Oligo(dT)25, using 3 rounds of isolation per the manufacturer’s protocol. RNA libraries were prepared with 10–50 ng purified mRNA using NEBNext Ultra directional RNA library Prep Kit for Illumina (New England BioLabs, Ipswich, MA, USA) following the manufacturer’s protocol. The library size was approximately 450 bp as measured using 2100 Bioanalyzer with Agilent High Sensitivity DNA kit (Agilent). Concentrations of the samples were measured using Qubit dsDNA HS kit (ThermoFisher) on a Qubit fluorometer. A total of 48 libraries were combined into two pooled samples at a final concentration of 1.8 pM each and sequenced using an Illumina NextSeq 500 sequencer in high output mode, providing 909 million paired-end (PE) reads (2 × 150 bp). 

### 2.4. RNA Sequencing, Alignment and Data Analysis

Because maize and fungal RNA were both sequenced, the reads were competitively aligned to the *Z. mays* B73 genome (version 4.35 from Gramene) and *A. flavus* NRRL 3357 genome (JCVI-afl1-v2.0; GCA_000006275.2). STAR (version 2.7.3a) was used to align the reads with the following settings: --alignIntronMax 60000 --outFilterMismatchNoverReadLmax 0.15 -outFilterMismatchNmax 23 -outFilterMultimapNmax 20 -twopassMode Basic. Reads mapping to genes were counted using featureCounts (version 1.5.2) [16]. with the settings: “-a -p -s 2 –primary”. 

### 2.5. Whole Genome Network Co-Expression Analysis (WGNCA) and Gene Selection

Co-expression networks were individually created using the variance stabilized mRNA counts from DESeq2 as input for WGNCA [17]. The network adjacency matrix was created with the settings “corFnc = ‘bicor’, type = ’signed hybrid’, power = 8”. To identify putative transcription factors, a list of InterPro IDs representing fungal transcription factor DNA binding domains from Shelest [18] was used to identify putative transcription factors in *A. flavus*. The full set of *A. flavus* InterPro annotations was generated using InterProScan version 5.19. Most transcription factors were selected for generating knockouts based on a combination of having (a) relatively high edge values, (b) high module membership values, (c) high positive correlation with growth on live corn kernels, and (d) a high number of edges (Table S1). The transcription factors demonstrated different expression levels when grown on live kernels relative to levels when grown on corn agar (Figure S1). 

### 2.6. Construct Assembly and Fungal Transformation

Gene knockout was performed using the established CRISPR/Cas9 technology [19]. DNA fragments that encoded single guide RNAs for targeting genes of interest were PCR amplified using pAf-CRISPR-yA (Addgene plasmid #191015) as the template. The PCR fragments were cloned into pAsp-AMA-gpdA-ptr (Addgene plasmid #191016) to generate gene targeting vectors for fungal transformation.

Preparation of protoplasts and fungal transformation using a polyethylene glycol/calcium chloride-mediumted protocol were carried out as previously described with minor modifications [20]. Selected primary pyrithiamine-resistant *A. flavus* transformants were transferred onto potato dextrose agar (PDA; Difco) plates and grown at 30 °C for two to three days. These were used for direct PCR and sequence analyses to confirm indel defects in the targeting genes. 

For sequencing to confirm mutagenesis, direct PCR using fungal mycelia as genomic templates and primers that encompassed targeted sequences was performed with a Phire Plant Direct PCR Master Mix (ThermoFisher Scientific). The PCR protocol consisted of an initial denaturation at 98 °C for 5 min, follow by 40 cycles of denaturation at 98 °C for 5 s, annealing at 60 °C for 20 s/kb, and extension at 72 °C for 30 s [21]. Purified PCR products were sequenced at the Genomics and Bioinformatics Research Unit of the Agricultural Research Service, US Department of Agriculture (ARS, Stoneville, MS, USA).

### 2.7. Growth Rate and Spore Production by Transcription Factor Knockout Strains

To measure growth rates of each transcription factor mutant, CA14 control and mutant strains were grown on PDA to obtain spore suspensions to be used as inoculum. Twenty microliters of 1 × 10^6^ spore suspension was center point inoculated on PDA and YGT agar plates and incubated at 30 °C in 12 h light/dark cycles. Growth was then measured at 3, 4, 5, 6, and 7 days of incubation. Two perpendicular measurements were taken for each plate. Seven-day old spores were manually harvested using four 12 mL aliquots of 0.02% Triton X-100, depending on the production of spores. An Olympus R1 cell counter (Olympus Corp, Tokyo, Japan) was used to determine relative spore counts per unit volume. All experiments were performed in triplicate. 

A linear model estimated growth rate differences between *A. flavus* knockout mutants with SAS version 9.4 (SAS Institute, Cary, NC, USA). The response variable was colony diameter, continuous effect was colony age, and the fixed effects were medium, mutant and their interactions. A linear model estimated analysis of variance with SAS (SAS Institute, Cary, NC, USA) to determine if there were differences in spore productions among the knockout mutants. The response variable was spores per mL and fixed effect was *A. flavus* knockout mutant. Means for each knockout mutant were compared to CA14 with post-hoc comparisons of least square means (LS-means). 

### 2.8. Aflatoxin and Cyclopiazonic Acid Analysis

Following growth of fungal strains on PDA medium or maize kernels (see Section 2.2), aflatoxin and cyclopiazonic acid CPA were extracted for analysis. Pulverized maize samples (1 g) and PDA agar were extracted with 5 mL methanol on shaker (85 rpm) for 12 h in the dark. All experiments were performed in triplicate. The extracts were filtered through cotton plugs. The filtrates were concentrated on a speedvac (Savant, Thermo Scientific, Waltham, MA, USA). Each extract was re-dissolved in methanol (1 mL), particulates were removed via centrifuge. A portion of the extract was diluted 100-fold for aflatoxin analysis. Analysis was performed on a Waters (Waters Corp., Milford, MA, USA) ACQUITY UPLC system (40% methanol in water, BEH C18 1.7 μm, 2.1 mm × 50 mm column) using fluorescence detection (Ex = 365 nm, Em = 440 nm). An aflatoxin B_1_ (AFB_1_) analytical standard (Sigma-Aldrich, St. Louis, MO) was used to identify and quantify aflatoxin content, which was expressed in ng AF/g corn sample.

To assess the presence of cyclopiazonic acid, the re-dissolved, centrifuged extracts were diluted 10-fold and analyzed on a Waters Acquity UPLC and Xevo G2 XS QTOF mass spectrometer (MS). The MS was equipped with a Z-spray ionization source running in ESI+ mode using Waters MassLynx 4.2 software (source temperature: 100 °C; desolvation temperature: 250 °C; desolvation gas flow: 600 L/h; cone gas flow: 50 L/h; capillary voltage: 3.0 kV; sampling cone voltage: 40 V). Analyses were performed in sensitivity and continuum mode, with a mass range of *m*/*z* 50–1200 and a scan time of 0.1 s. A data-independent acquisition method with elevated collision energy (MS^E^) was used with 6 eV low energy and a high energy ramp from 15−45 eV. Separation was achieved with a gradient solvent system (A: 0.1% formic acid in water; B: 0.1% formic acid in acetonitrile) on a Waters BEH C18 1.7 µm, 2.1 × 50 mm column: 5% B (0–1.25 min.), to 25% B (1.25–1.5 min.), to 100% B (1.5−5.0 min.), then 100% B (5.0−7.5 min.), followed by column equilibration at 5% B (7.6–10.1 min.). Data were analyzed on Waters UNIFI 1.9.4 software using the “Quantify Assay Tof 2D” analysis method with lock mass corrected by UNIFI. CPA was purchased from Sigma-Aldrich (Sigma-Aldrich, St. Louis, MO, USA) and used for quantification. CPA content was expressed in ng CPA/g corn sample.

Three separate linear models estimated analysis of variance with SAS (Cary, North Carolina) to determine if there were differences in aflatoxin on PDA and maize and CPA production on maize among the knockout mutants. The response variables were aflatoxin (ppb) or CPA (ppb) and fixed effect was *A. flavus* knockout mutant. Aflatoxin and CPA means for each knockout mutant were compared to CA14 with post-hoc comparisons of least square means (LS-means). A 0.1 constant was added to all CPA measurements to account for over-dispersion resulting from zero values.

### 2.9. Menadione-Induced Oxidative Stress Response

*A. flavus* spores were point inoculated on 2XV8 medium (10% V8 juice, 0.2% uracil, 0.26% ammonium sulfate, 2.0% agar; pH 5.2) and grown for two days at 30 °C under constant light. Spore stocks were made by taking one agar plug from a fungal colony on the surface of a 2XV8 plate using Spectrum transfer tubes 153 × 6 mm and transferring into 1 mL of 0.01% Triton buffer. Spores were vortexed for 15 seconds and plated onto PDA and Wickerham Agar (W; 2 g yeast extract, 3 g peptone, 2 g dextrose, 30 g sucrose, 5 g corn steep, 2 g NaNO_3_, 0.7 g K_2_HPO_4_ anhydrous, 0.5 g MgSO_4_ • 7H2O, 0.2 g KCl, 10 mg FeSO_4_ • 7H2O, 1.5% agar; pH 5.5) both supplemented with 0.2% uracil. Spores were also inoculated on PDA+uracil and W+uracil agar plates with the addition of 0.1 mM and 0.2 mM menadione dissolved in 95% ethanol to study oxidative stress response. All plates were incubated at 30 °C in the dark for 5 days.

## 3. Results

### 3.1. Morphology of Transcription Factor Knockout Strains

#### 3.1.1. General Morphology

**Figure 1:**
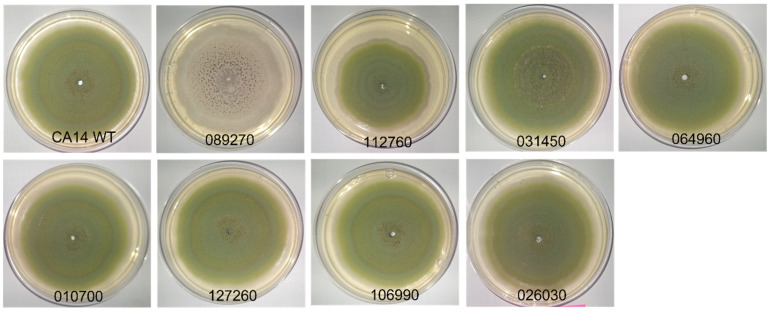
*A. flavus* knockout strains with the indicated transcription factors deleted were grown on PDA for 7 days at 30 °C under 12 h light/dark cycles. CA14 positive control shows a wild-type phenotype of green pigmentation associated with spore production. AFLA_089270 exhibits a lack of green pigmentation associated with spore production in CA14.

As described above, whole genome network correlation analysis of RNA sequencing data was used to identify core transcription factors potentially involved in *A. flavus* virulence. These transcription factors were selected based on several factors, including the presence of DNA-binding domains, their association with the trait of actively infecting corn (as opposed to growing on medium), and due to sharing gene expression patterns with a relatively high number of genes (high edge values) (Supplemental Figure S1 and Supplemental Table S1). To examine the morphological changes induced by knockout of the putative virulence-related transcription factors (Supplemental Figure S2), we plated the mutant strains and a CA14 positive control strain on both PDA (Figure 1) and YGT medium (data not shown) in triplicate. The representative images in Figure 1 illustrate that most knockout strains maintained the characteristic morphology of L strain *A. flavus* isolates observed in the CA14 control strain. The AFLA_089270 knockout strain exhibited a significant reduction in spore production compared to the control. The morphological features were very similar in YGT and PDA medium.

#### 3.1.2. Growth Rate of Transcription Factor Knockout Strains

**Figure 2:**
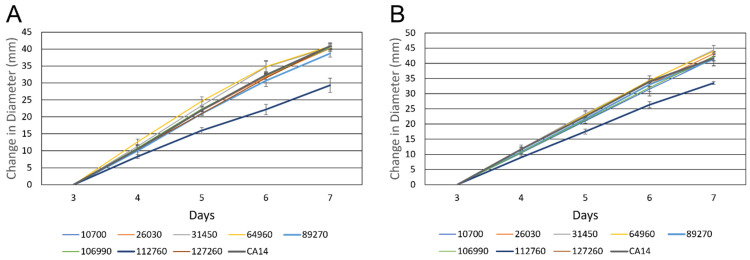
Growth curves of the *A. flavus* transcription factor knockout strains on (**A**) YGT medium and (**B**) PDA medium. Radial growth over 5 days (starting on day 3) was measured for each of the transcription factor knockout strains. All strains exhibit similar growth rates relative to the control CA14 strain except AFLA_112760, which exhibited decreased growth rates relative to the control strain. Error bars represent the standard deviation.

To determine if knocking out the transcription factors impacted growth rates, spore suspensions of equal concentrations were center-plated onto PDA plates in triplicate and grown for 7 days. The results indicate that knocking out the putative transcription factor AFLA_112760 (T_250_ = −11.22, *P* < 0.0001) resulted in reduced growth rate. All other knockout strains maintained growth rates comparable to strain CA14 (Figure 2).

#### 3.1.3. Aflatoxin, Spore Production on Agar Medium

**Figure 3:**
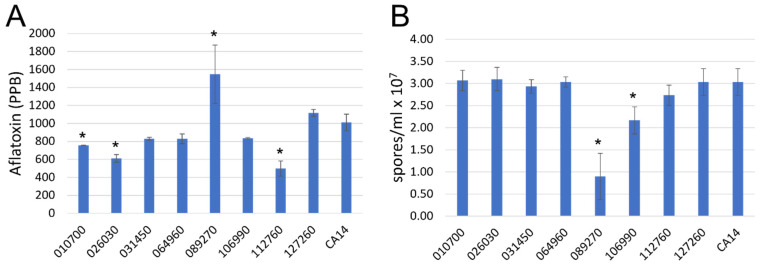
Transcription factor knockout strains exhibit altered sporulation and aflatoxin production. (**A**) Aflatoxin extracted from PDA medium after 7 days of growth from the respective transcription factor knockout strains. (**B**) Spore counts obtained from transcription factor knockout strains grown on PDA. Error bars represent the standard deviation. Asterisks (*) represent different LS means from CA14 at α < 0.05.

Aflatoxin production on PDA plates was significantly higher in AFLA_089270 knockout strain (T_18_ = 2.76, *P* = 0.0135) as compared to the CA14 strain, and the AFLA_112760 (T_18_ = −2.11, *P* = 0.0502), AFLA_010700 (T_18_ = −2.22, *P* = 0.0400), AFLA_026030 (T_18_ = −2.62, *P* = 0.0179) knockout strains were significantly reduced. Examination of spore concentrations of each knockout strain showed that the AFLA_089270 (T_18_ = −9.29, *P* < 0.0001) knockout strain and AFLA_106990 (T_18_ = −3.77, *P* = 0.0014) to a lesser extent had significant reduction (ANOVA, described above) in spore production (Figure 3). All other knockout strains maintained a wildtype level of spore production.

### 3.2. Ultra-High Pressure Liquid Chromatography (UPLC) Analysis of Aflatoxin and Cyclopiazonic Acid Levels

**Figure 4:**
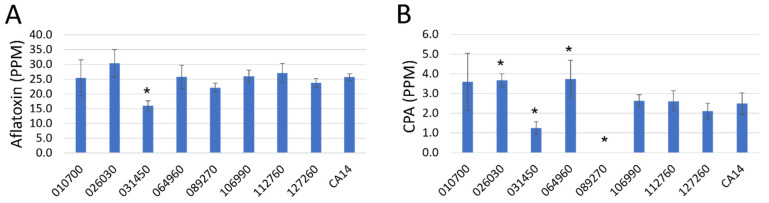
Aflatoxin (**A**) and cyclopiazonic acid (CPA) (**B**) levels present in maize kernels after 7 days of infection with the indicated transcription factor knockout strains. Error bars represent the standard deviation. Asterisks (*) represent different LS means from CA14 at α < 0.05.

To determine if knocking out the transcription factors had a measurable effect on aflatoxin or CPA accumulation in maize kernels, we inoculated kernels with CA14 control and mutant spores then allowed infection to proceed for 7 days. UPLC analysis determined that there was significantly less aflatoxin accumulation in the kernels in the AFLA_031450 (T_18_ = −3.60, *P* = 0.0021) knockout strain (Figure 4A). The same UPLC analysis also determined that CPA levels were reduced in this strain (T_18_ = −2.25, *P* = 0.0370) (Figure 4B). AFLA_089270 knockout strain showed no detectable levels of CPA accumulation in the kernels.

### 3.3. Menadione Oxidative Stress Assays

**Figure 5:**
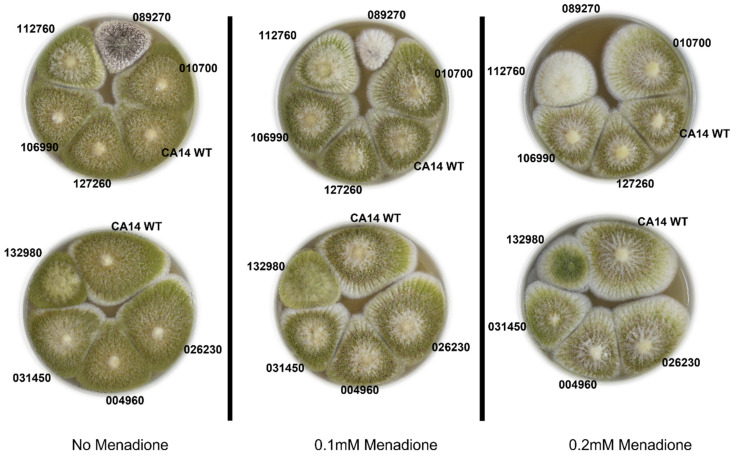
Menadione exposure to *A. flavus* transcription factor knockout strains indicates low tolerance to oxidative stress in two strains, transcription factor knockout AFLA_089270 and AFLA_112760. The two plates vertical to each other contain all the strains tested, with increased menadione concentration from left to right.

Because the virulence of fungal pathogens is in part dependent on their response to the “oxidative burst” of reactive oxygen species (ROS) produced by the host plant as a defense mechanism, we tested the transcription factor knockout strains for their ability to survive on agar supplemented with menadione. The AFLA_089270 knockout strain exhibited the largest response to increased menadione, with a highly reduced capacity to grow at 0.1 mM menadione, and complete loss of growth at 0.2 mM menadione (Figure 5). In comparison, the AFLA_112760 knockout strain also exhibited inhibited growth, however to a lesser extent than AFLA_089270 knockout strain. The remaining transcription factor knockout strains retained their ability to mitigate the highly oxidative environment and maintained their characteristic phenotype.

## 4. Discussion

In this study, we selected a panel of putative virulence-related transcription factors from co-expression analysis of RNA-sequencing data, and generated knockout strains for these transcription factors in a CA14 strain of *Aspergillus flavus*. The knockout strains were analyzed for morphological development, secondary metabolite biosynthesis and oxidative stress-response to elucidate which transcription factors potentially contribute to *A. flavus* virulence while infecting live kernels. These experiments revealed three transcription factors, two of which are novel and uncharacterized, and implicate their involvement in maize seed infection. The third gene analyzed is necessary for CPA biosynthesis and is menadione sensitive, suggesting an oxidative stress response-related mechanism for its role in pathogenicity.

AFLA_112760 is an uncharacterized C2H2 zinc finger domain containing protein showing high conservation across the Asgergilli and related genera such as *Penicillium* and *Talaromyces*. Our data showing reduced growth rate and reduced growth in menadione assays suggest a potential role in virulence, although a decrease in aflatoxin levels in our infection assay was not observed. AFLA_031450, a CP2 transcription factor, did demonstrate reduced aflatoxin accumulation in kernels, indicating reduced pathogenicity, especially given that we did not see reduced growth rates on medium for this strain. A Blastp search indicates that this gene is currently hypothetical and uncharacterized, although it has high sequence identity with the grainyhead-like family of transcription factors, of which the function appears mostly unknown in fungi. Microarray assays of grainyhead-like gene knockout mutants in *Neurospora* did identify potential regulation of genes involved with fungal virulence such as the metalloprotease *MEP1* and exo-beta 1,3 glucanase genes [22].

Our results showed that the transcription factor AFLA_089270 knockout strain exhibited slightly decreased (although not statistically significant) aflatoxin accumulation in the kernel infection assays, no cyclopiazonic acid production, reduced spore production on medium, and reduced growth in menadione assays. The high levels of AF production on PDA relative to kernels could be due to high sugar content of the medium, possibly implicating a role in moderating or influencing substrate availability. This transcription factor, annotated as HacA, has been previously characterized in yeast, *A. flavus, A. niger, A. fumigates, A. oryzae* and *Trichophyton rubrum* [23,24,25,26,27]. These studies initially examined its role in regulating the cellular unfolded protein response (UPR), a complex signal-transduction mechanism for regulating endoplasmic reticulum function under periods of high demand, often resulting in misfolded protein accumulation. However, as in several other model systems, the previously mentioned gene knockout studies in *A. oryzae* and *A. flavus* identified diverse roles in development and secondary metabolism, including modulating the production of spores, aflatoxin production, and interestingly, the reduction of amylase genes, possibly offering additional clues into its role in fungal virulence. Earlier studies have further provided evidence linking HAC1 (the yeast analog to HacA) and an increase in anti-oxidative stress-related genes [28]. Finally, previous studies in *A. flavus* attempting to generate HacA knockouts generated only heterogenous knockout nuclei, making this the first study to our knowledge using HacA single-gene knockout strains to demonstrate the role of HacA in CPA production and oxidative stress in *A. flavus* [29].

Pathogen detection by a susceptible plant can result in an increase in the production of ROS as a host defense mechanism [30]. Infecting pathogens may employ two forms of defense against the host ROS production: one is the increased production of nonenzymatic antioxidants such as flavonoids, alkaloids, and carotenoids; another strategy involves active ROS scavenging by way of enzymes such as superoxide dismutase and catalases [31,32]. Previous data have indicated that the production of aflatoxins and other metabolites such as aflatrem likely act as a fungal counter response, and could even be dependent upon the presence of ROS [33,34]. To our knowledge, limited information has been provided regarding a potential role of CPA in plant pathogenesis; however, Chalivendra et al. [35] reported that *A. flavus* isolates infecting maize exhibit higher levels of CPA compared to isolates not obtained from maize, and knockout of CPA secondary metabolic cluster genes reduces *A. flavus* pathogenicity in maize. The data here further support a potential role of CPA in the oxidative stress response of *A. flavus*.

The current study suggests that three transcription factors identified in *A. flavus* are potential regulators of fungal virulence. One of these, the HacA gene, potentially does so by regulating CPA production thus mitigating the oxidative stress response of the fungus. Pursuing further characterization of the genes mentioned above has the high potential of making them suitable candidates for targeting *A. flavus* in aflatoxin and/or CPA mitigation strategies such as HIGS or other approaches targeting the pathogen.

## Data Availability

All data reported here is included.

## Supplementary Materials

The following supporting information can be downloaded at: https://www.mdpi.com/article/10.3390/jof9010118/s1, Figure S1: rlog counts of transcription factors; Figure S2: Details of knockout mutations; Table S1: Networking results for transcription factors.
