# Supplementary material for: Putative Core Transcription Factors Affecting Virulence in Aspergillus flavus during Infection of Maize
Source: J Fungi (Basel). 2023 Jan 14;9(1):118. doi: 10.3390/jof9010118 (PMC9861280; doi:10.3390/jof9010118)
Supplement: Supplementary file 1 [file jof-09-00118-s001.zip › Supp Figure S2.pdf]

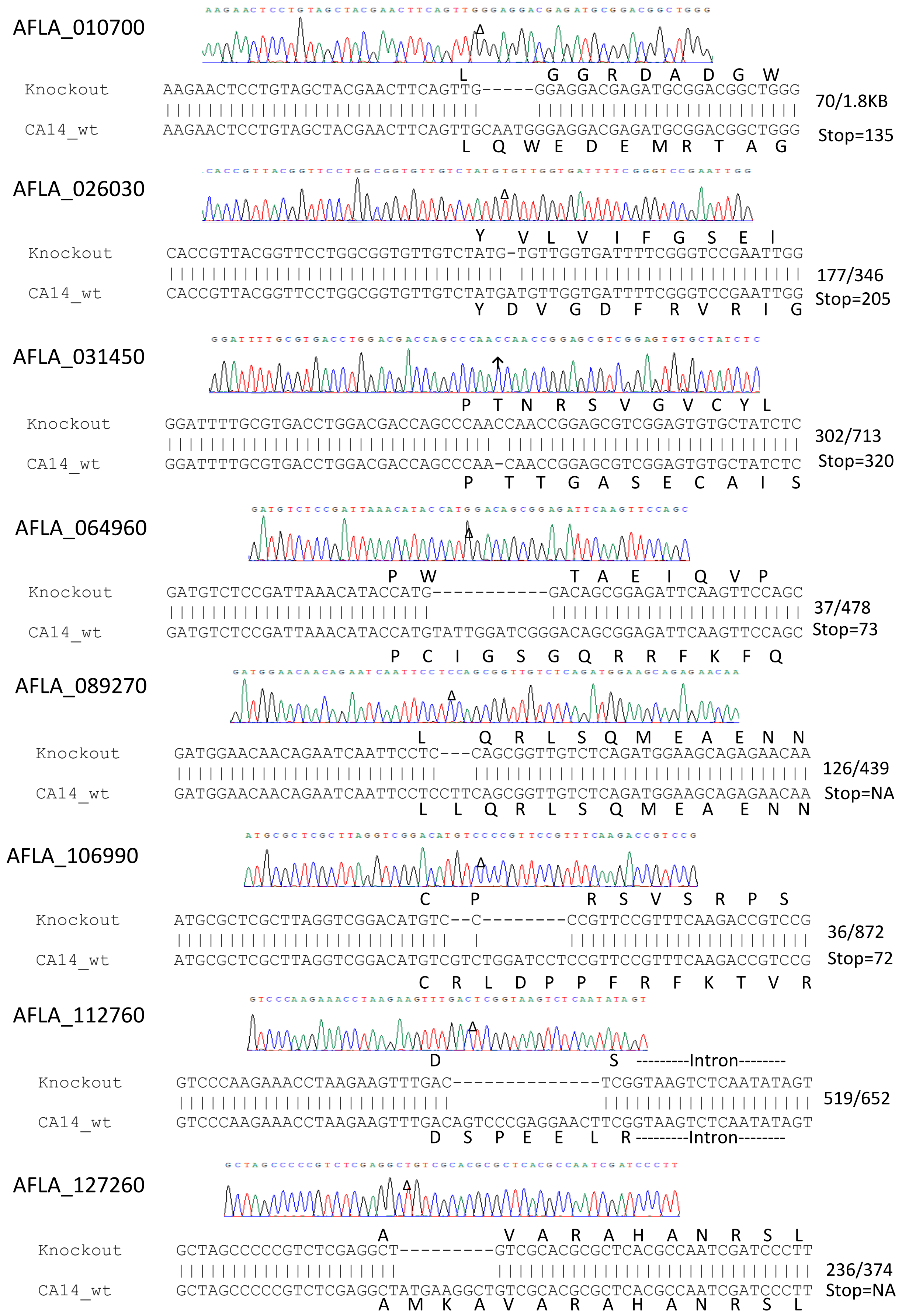

Supplemental Figure S2. Sequence defects of *A. flavus* gene knockouts. The triangle symbol indicates the position of deletion. The up arrow indicates a nucleotide insertion. Letters are amino acid translations of the coding sequence. Numbers at the end of each sequence indicate (aa position of mutation/total aa's in sequence). Location of premature stop codon indicated at end of sequence.
