# Supplementary figures and images for: Putative Core Transcription Factors Affecting Virulence in Aspergillus flavus during Infection of Maize
Source: J Fungi (Basel). 2023 Jan 14;9(1):118. doi: 10.3390/jof9010118 (PMC9861280; doi:10.3390/jof9010118)

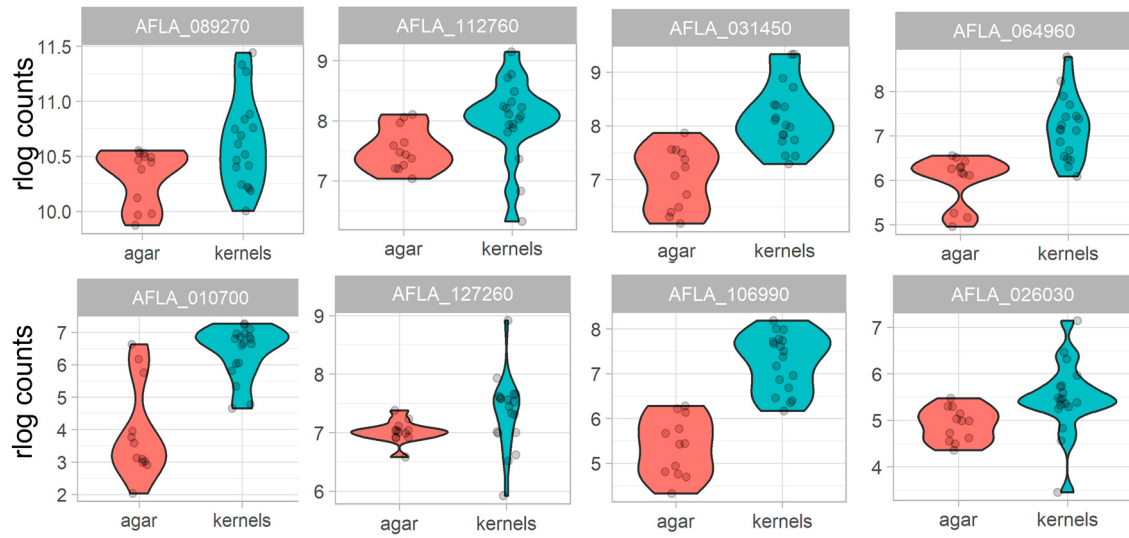

Figure S1: rlog counts of transcription factors.

Supplement: Supplementary file 1 [file jof-09-00118-s001.zip › Suppl Figure S1.pdf]
